# Anacardic acid, a histone acetyltransferase inhibitor, modulates LPS-induced IL-8 expression in a human alveolar epithelial cell line A549

**DOI:** 10.12688/f1000research.2-78.v1

**Published:** 2013-03-06

**Authors:** Tetsuo Yasutake, Hiroo Wada, Manabu Higaki, Masuo Nakamura, Kojiro Honda, Masato Watanabe, Haruyuki Ishii, Shigeru Kamiya, Hajime Takizawa, Hajime Goto

**Affiliations:** 1 Department of Respiratory Medicine, Kyorin University School of Medicine, Tokyo, 181-8611, Japan

## Abstract

**Objective and design: **The histone acetylation processes, which are believed to play a critical role in the regulation of many inflammatory genes, are reversible and regulated by histone acetyltransferases (HATs), which promote acetylation, and histone deacetylases (HDACs), which promote deacetylation. We studied the effects of lipopolysaccharide (LPS) on histone acetylation and its role in the regulation of interleukin (IL)-8 expression.

**Material: **A human alveolar epithelial cell line A549 was used *in* *vitro*.

**Methods:** Histone H4 acetylation at the IL-8 promoter region was assessed by a chromatin immunoprecipitation (ChIP) assay. The expression and production of IL-8 were evaluated by quantitative polymerase chain reaction and specific immunoassay. Effects of a HDAC inhibitor, trichostatin A (TSA), and a HAT inhibitor, anacardic acid, were assessed.

**Results:** *Escherichia coli*-derived LPS showed a dose- and time-dependent stimulatory effect on IL-8 protein production and mRNA expression in A549 cells *in vitro*. LPS showed a significant stimulatory effect on histone H4 acetylation at the IL-8 promoter region by ChIP assay. Pretreatment with TSA showed a dose-dependent stimulatory effect on IL-8 release from A549 cells as compared to LPS alone. Conversely, pretreatment with anacardic acid inhibited IL-8 production and expression in A549 cells.

**Conclusion:** These data suggest that LPS-mediated proinflammatory responses in the lungs might be modulated via changing chromatin remodeling by HAT inhibition.

## Introduction

Pneumonia is an important socio-medical problem and one of the leading causes of death in the world ^1^. Gram-negative rods, crucial pathogens in hospital- as well as community-acquired pneumonia, produce and release endotoxins which constitute lipopolysaccharides (LPS). Once inhaled via respiratory routes, LPS stimulate alveolar structural as well resident cells to release many kinds of bioactive agents such as proinflammatory cytokines into local microenvironments ^2^.

Recent studies have emphasized a crucial role for the lung epithelium as an important sentinel and effector system of innate immunity ^3– 5^. Upon infectious agents and their products being inhaled into the respiratory systems, activated lung epithelium may contribute to the regulation of the immune response as the first-line defense mechanism ^6^. Among those defense responses, it seems important that alveolar epithelial cells express and release a variety of pro-inflammatory cytokines and chemokines into alveolar microenvironments ^6, 7^. A CXC chemokine, interleukin (IL)-8, plays an important role in the acute recruitment of immune/inflammatory cells, especially neutrophils, to the site of infection in the lung ^8, 9^. LPS bind to Toll-like receptors (TLR) and thereby activate downstream signal transduction pathways which ultimately phosphorylate cytosolic I-κB kinase ^10, 11^. Then, I-κB is phosphorylated to induce free-form of NF-κB, which translocates into the nucleus. The NF-κB binds to its specific binding sites on the promoter regions and enhances the expression of IL-8 gene ^12, 13^.

Increasing evidence has indicated that the expression of many inflammatory genes involves the remodeling of the chromatin structure provided by histone proteins ^14, 15^. Remodeling of chromatin within the nucleus is controlled by the degree of acetylation/deacetylation of histone residues on the histone core around which DNA is coiled. Histone acetylation results in the unwinding of the chromatin structure, which enhances the binding of transcription factors to their specific promoter sites on the DNA ^16^. Nuclear histone acetylation is a reversible process and is regulated by a group of histone acetyltransferases (HATs) which promote acetylation, and histone deacetylases (HDACs) which promote deacetylation ^17, 18^. The loosening of DNA-histone interactions and the subsequent unmasking of transcription factor binding sites is controlled by specific covalent modifications of accessible N-terminal histone tails ^19^. Among the four core histone proteins that comprise the central chromatin core (H2A, H2B, H3, and H4), acetylation processes on H3 and H4 seem particularly important in gene regulation. For example, Gilmour and associates ^20^ found that acetylation on H4 played an important role in environment particle-induced IL-8 production in A549 cells. Viable *Listeria monocytogenes*-stimulated endothelial cells showed increased expression of IL-8, and that process depended on modifications of H3 and H4 ^21^. Although the host response in pneumonia is characterized by massive cytokine production, and altered histone modifications have been observed in diseased lungs ^22^, it is not fully elucidated how histone modifications contribute to innate immune regulation in the lung.

In this study, we tried to determine whether *Escherichia coli*-derived LPS, one of the mainstream stimuli upon bacterial respiratory infection, altered histone acetylation/deacetylation balance, and to see whether the modulation of HDACs or HATs by their specific inhibitors (i.e. trichostatin A [TSA] for HDACs and anacardic acid for HATs) affected IL-8 gene expression and protein production in an alveolar epithelial cell line A549 *in vitro*.

## Materials and methods

### Cell culture and stimulation

Human alveolar epithelial cell line A549 was obtained from the American Type Culture Collection (ATCC, Manassas, VA, USA) via DS Pharma Biomedical Co., Ltd (Tokyo, Japan). A549 cells were cultured in Dulbecco’s Modified Eagle’s Medium (Sigma-Aldrich, St. Louis, MO, USA) containing 10% fetal bovine serum (FBS) (Invitrogen, Grand Island, NY, USA) and 1% penicillin-streptomycin (Sigma-Aldrich, St, Louis, MO, USA), and incubated at 37°C in 5% CO _2_ atmosphere. Cells were cultured to 80% confluency as judged under inverted microscopy before the medium was replaced with serum-free Dulbecco’s Modified Eagle’s Medium and incubated for a further 15 h. The cells were stimulated with different concentrations of *E.coli*-derived LPS (026:B6, Sigma-Aldrich, St, Louis, MO, USA) for further experiments. To evaluate the effects of HDAC and HAT inhibitors, cells were pre-treated with TSA or anacardic acid (Sigma-Aldrich, St, Louis, MO, USA; dissolved in dimethyl sulfoxide [DMSO] and further diluted for use) at the concentrations indicated, 1h prior to stimulation with LPS (10 μg/ml).

### Quantitative reverse transcription polymerase chain reaction (qRT-PCR)

Total RNA from A549 cells was isolated with the RNeasy Mini kit (Qiagen, Hamburg, Germany), and cDNA was prepared using the Im Prom II reverse transcription system (Promega, Madison, WI, USA) for reverse transcription, and all procedures were conducted according to the manufacturers’ instructions. Gene transcript levels of IL-8, and glyceraldehyde-3-phosphate dehydrogenase (GAPDH) were quantified by real-time PCR using a reaction mixture with SYBR Premix Ex Taq (Takara, Tokyo, Japan) on a 7500 real-time PCR system (Applied Biosystems, Foster City, CA, USA). The primer sets for IL-8 were: (forward) 5’-CTGATTTCTGCAGCTCTGTG-3’; (reverse) 5’-TTCACTGGCATCTTCACTG-3’ and for GAPDH were: (forward) 5’-TGAACGGGAAGCTCACTGG-3’ (reverse) 5’-TCCACCACCCTGTTGCTGTA-3’ The relative amount of gene transcript was estimated after normalization by dividing the calculated value for the gene of interest by the GAPDH value.

### Chromatin immunoprecipitation assay (ChIP assay)

Chromatin immunoprecipitation was performed using Millipore’s ChIP kit (Billerica, Massachusetts, USA) with an acetyl-histone H4 antibody. Cells were cultured to 80% confluency in 100mm culture plates. Cells were cross-linked by adding 1% formaldehyde for 10 minutes at room temperature in shaking. Then, 1ml of 10×glycine was added to 10ml of growth media to each dish to quench unreacted formaldehyde at room temperature for 5 minutes. Cells were washed twice with cold 10ml phosphate buffered saline (PBS). One ml PBS containing 1×protease inhibitor cocktail II prepared in the ChIP kit was added to each dish. Cells were scraped from each dish into a conical tube. The tubes were centrifuged at 700×g at 4°C for 5 minutes to pellet cells. Each cell pellet was resuspended in 1ml of SDS lysis buffer containing 1×protease inhibitor cocktail II prepared in the ChIP kit. Chromatin was sonicated to an average DNA length of 200–1000 bp using an Ultrasonic Disruptor UD-200 sonicator (TOMY, Tokyo, Japan). Sonicated samples were centrifuged and the supernatant was collected. Samples (100μl) of the extracted chromatin were diluted with 900μl ChIP Dilution Buffer (0.01% SDS, 1.1% Triton X-100, 1.2mM EDTA, 16.7mM Tris-HCl, pH 8.1, 167mM NaCl), precleared (1 hour) by incubation with 60μl Protein G Agarose containing 1×protease inhibitor cocktail II, and subjected to immunoprecipitation with a specific antibody with rotation overnight at 4°C. The antibody used for ChIP assays was anti-acetyl histone H4 antibody (Millipore, Billerica, Massachusetts, USA). Immunocomplexes were collected by adsorption onto 60μl Protein G Agarose and the beads were washed five times sequentially with Low Salt Immune Complex Wash Buffer (0.1% SDS, 1% Triton X-100, 2mM EDTA, 20mM Tris-HCl, pH 8.1, 150mM NaCl), High Salt Immune Complex Wash Buffer (0.1% SDS, 1% Triton X-100, 2mM EDTA, 20mM Tris-HCl, pH 8.1, 500mM NaCl) and LiCl Immune Complex Wash Buffer (0.25M LiCl, 1% IGEPAL CA630, 1% deoxycholic acid (sodium salt), 1mM EDTA, 10mM Tris, pH 8.1), which were prepared in the ChIP kit. Precipitates were washed twice with TE Buffer (10mM Tris-HCl, pH 8.0, 1mM EDTA), and antibody-chromatin fragments were eluted from the beads with 1% sodium dodecyl sulphate in 0.1 M NaHCO _3_. Cross-links were reverted by adding 200mM NaCl and heating at 65°C for 5 hours. In total, 10mg/ml RNase A were added and samples were then incubated for 30 minutes at 37°C. 10mg/ml proteinase K, 10mM EDTA and 40mM Tris-HCl were added and samples were then incubated for 2 hours at 45°C.

A total of 1ml of Bind Reagent A was added to each sample, and mixed well by pipetting. The sample was transferred up to a spin filter placed in a collection tube, and was centrifuged for 30 seconds at 12000×g. Then, 500μl of Wash Reagent B was added to each spin filter placed in a collection tube, and was centrifuged for 30 seconds at 12000×g. Then, 50μl of Elution Buffer C was added to each spin filter placed in a clean collection tube, and was centrifuged for 30 seconds at 12000×g to recover purified DNA. The IL-8 enhancer regions were quantified by real-time PCR using a reaction mixture with SYBR Premix Ex Taq (Takara, Tokyo, Japan) on a 7500 real-time PCR system (Applied Biosystems, Foster City, CA, USA). The primer sets for IL-8 were: (forward) 5’- CAGAGACAGCAGAGCACAC-3’; (reverse) 5’- ACGGCCAGCTTGGAAGTC-3’. All PCR signals from immunoprecipitated DNA were normalized to PCR signals from non-immunoprecipitated input DNA.

### ELISA for IL-8 measurement

The concentration of IL-8 in the media was determined by sandwich ELISA kit (R&D Systems, San Diego, CA) according to the manufacturer’s instructions.

### Statistical analysis

Data were analyzed with the Statistical Package for Social Science (SPSS) version 17.0 for Windows (SPSS Inc., Chicago, IL, USA). Values are expressed as mean ± standard error (SE). Mann-Whitney U test was performed for comparisons between groups. A P-value < 0.05 was considered significant. All P-values were two-sided.

## Results

### LPS stimulated production of IL-8 in A549 cells

**Figure 1.**
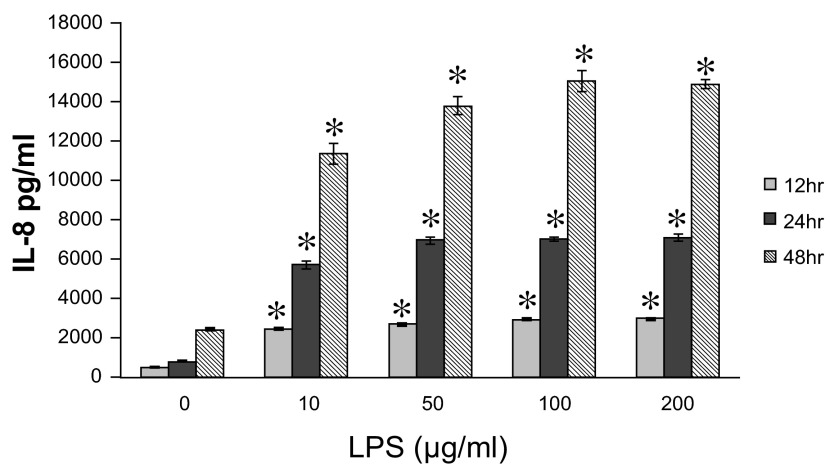
The dose- and time- dependent interleukin-8 (IL-8) release from A549 cells *in vitro*. A549 cells were cultured until 80% confluence and stimulated by different concentrations of lipopolysaccharide (LPS) (10 ~ 200μg/ml). LPS showed a time- and dose-dependent stimulatory effect on IL-8 release at each concentration. Data were expressed in mean±SEM. *P<0.05, compared to LPS unstimulated cells at each concentration and time point (Mann-Whitney U test), n=4.

We analyzed IL-8 production in LPS stimulated A549 cells. A549 cells were stimulated by different concentrations of LPS (10 ~ 200μg/ml) for indicated time periods. As shown in Figure 1, LPS showed a time- and dose-dependent stimulatory effect on IL-8 release.

### LPS induced expression of IL-8 gene in A549 cells

**Figure 2.**
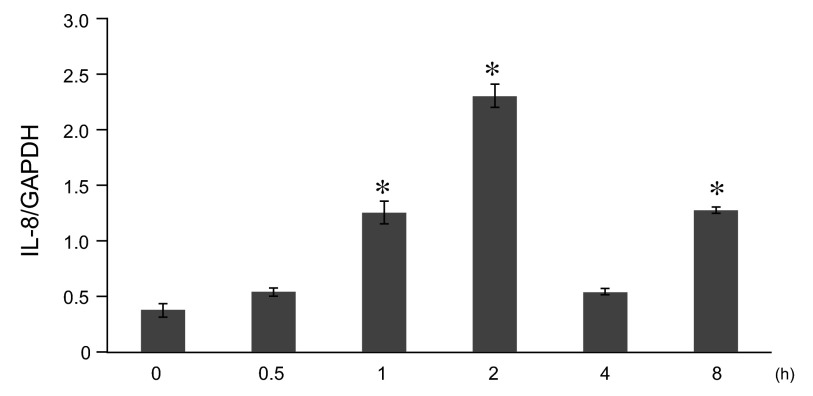
Time course analysis of lipopolysaccharide (LPS)-mediated interleukin-8 (IL-8) gene expression in A549 cells *in vitro*. A549 cells were cultured until subconfluence and stimulated by LPS at 10μg/ml. After 0 ~ 8 hrs, IL-8 mRNA expression was analyzed by quantitative reverse transcription polymerase chain reaction (qRT-PCR). The IL-8 mRNA levels were normalized to glyceraldehyde-3-phosphate dehydrogenase (GAPDH) levels. Data were expressed in mean±SEM. *P<0.05: compared to LPS 0hr stimulated cells (Mann-Whitney U test), n=4.

A549 cells were stimulated by 10μg/ml LPS, and the time course in levels of IL-8 mRNA was analyzed by qRT-PCR. IL-8 mRNA levels showed a gradual increase in response to LPS, reaching a maximum level 2 h after initial stimulation with 10μg/ml, which then decreased after that point ( Figure 2).

### LPS induced histone acetylation in A549 cells

**Figure 3.**
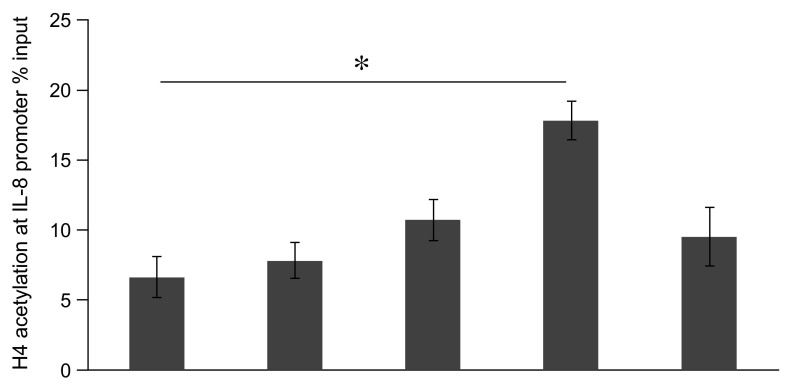
Time course analysis of lipopolysaccharide (LPS)-induced histone H4 acetylation at the promoter region of the interleukin-8 (IL-8) gene in A549 cells *in vitro*. A549 cells were stimulated 0 ~ 3 hr with LPS (10μg/ml). The results presented are from ChIP analyses using anti-acetyl H4 antibodies. All PCR signals from immunoprecipitated DNA were normalized to PCR signals from non-immunoprecipitated input DNA. Results are expressed as percentage of the input. Data were expressed in mean±SEM. *P<0.05: compared to LPS 0hr stimulated cells (Mann-Whitney U test), n=4.

Based on the previous reports showing that modifications of histones, in particular acetylation of H4, seem to contribute to the regulation of inflammatory genes such as IL-8 ^20^, we assessed acetylation of H4 after stimulation of LPS in A549 cells. We analyzed histone modifications at the IL-8 gene promoter by ChIP assay. A549 cells were stimulated with LPS (10μg/ml) for 0 min to 3 h. LPS induced a time-dependent increase of acetylation of H4 at the IL-8 promoter, and this increase peaked after 1 h (P<0.05, Mann-Whitney U test), and then decreased after 3 h of LPS stimulation ( Figure 3).

### Histone acetylation regulates IL-8 expression in LPS-stimulated A549 cells

**Figure 4.**
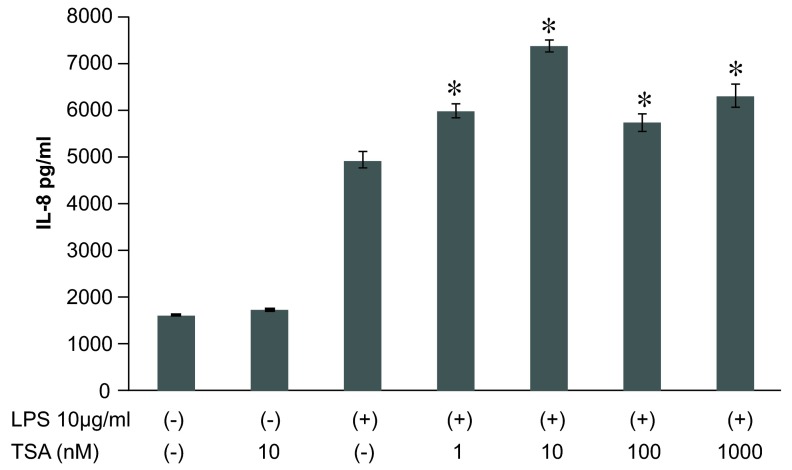
Effect of trichostatin A (TSA) on lipopolysaccharide (LPS)-stimulated interleukin-8 (IL-8) release from A549 cells *in vitro*. TSA at 1 ~ 1000nM treated 1 hr before LPS stimulation (10μg/ml) showed a significant stimulatory effect on LPS-induced IL-8 release. Data were expressed as mean±SEM. *P<0.05: compared to LPS-stimulated cells (Mann-Whitney U test), n=4.

**Figure 5.**
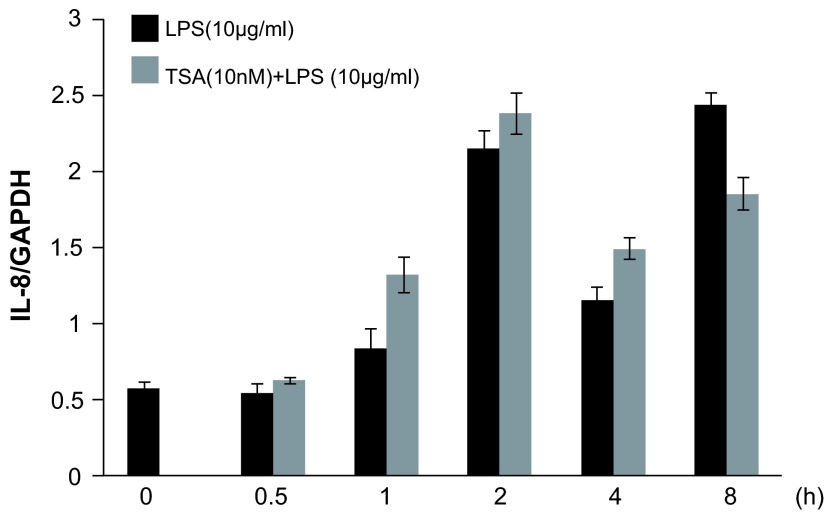
Time course analysis of trichostatin A (TSA) on lipopolysaccharide (LPS)-stimulated interleukin-8 (IL-8) gene activation from A549 cells *in vitro*. TSA at 10nM treated 1 h before LPS stimulation (10μg/ml) tended to show a stimulatory effect on LPS-induced IL-8 gene activation. After 0 ~ 8 h, the levels of IL-8 mRNA were analyzed by quantitative reverse transcription polymerase chain reaction (qRT-PCR). The IL-8 mRNA levels were normalized to glyceraldehyde-3-phosphate dehydrogenase (GAPDH) levels, n=4.

Next, we wondered whether inhibition of HDACs by TSA or blocking of HATs by anacardic acid impacts on LPS-induced IL-8 expression. We increased global histone acetylation by incubation of A549 cells with TSA (10nM, 24 h) which did not induce IL-8 secretion per se ( Figure 4). Preincubation for 1 h with TSA (10nM) before subsequent treatment with LPS significantly increased IL-8 release as compared to LPS alone ( Figure 4). Pretreatment with TSA (10nM) showed a tendency to increase IL-8 mRNA levels as assessed by qPCR analysis, but did not reach statistical significance ( Figure 5).

**Figure 6.**
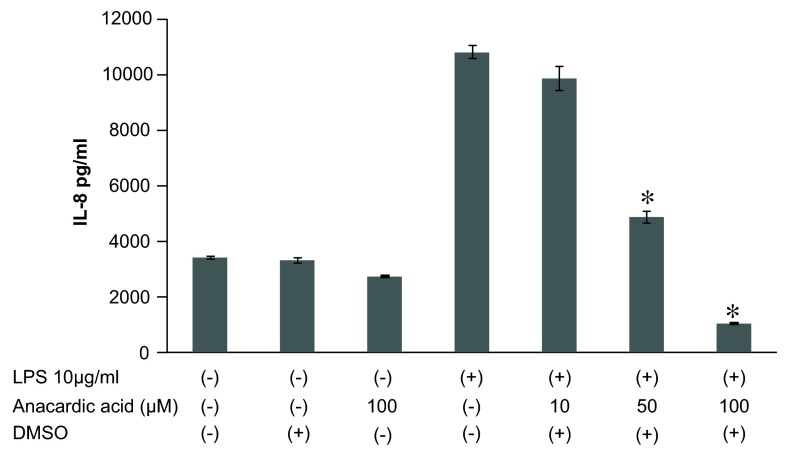
Effect of anacardic acid on lipopolysaccharide (LPS)-stimulated interleukin-8 (IL-8) release from A549 cells *in vitro*. Anacardic acid at 10 ~ 100μM treated 1 hr before LPS stimulation showed a significant inhibitory effect on LPS-induced IL-8 release. Data were expressed as mean±SEM. DMSO: dimethyl sulfoxide used for solvent. *P<0.05: compared to LPS (10μg/ml) stimulated cells (Mann-Whitney U test), n=4.

**Figure 7.**
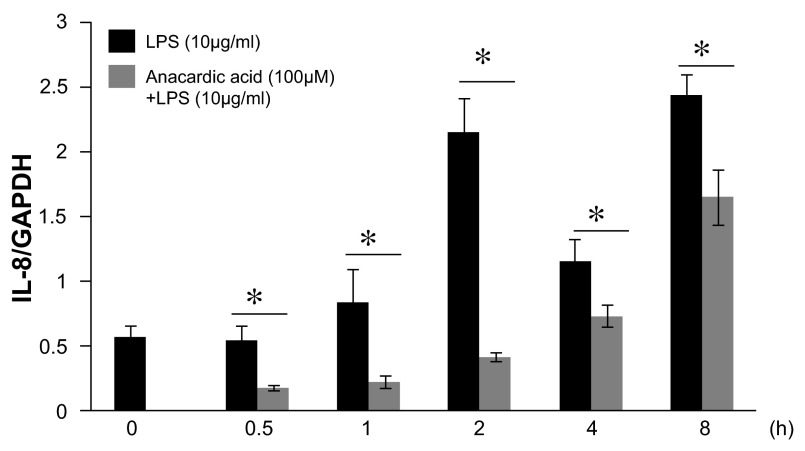
Time course analysis of anacardic acid on lipopolysaccharide (LPS)-stimulated interleukin-8 (IL-8) gene activation in A549 cells *in vitro*. Anacardic acid at 100μM treated 1 hr before LPS (10μg/ml) stimulation showed a significant inhibitory effect on LPS-induced IL-8 gene activation. After 0 ~ 8hrs, the levels of IL-8 mRNA were analyzed by quantitative reverse transcription polymerase chain reaction (qRT-PCR). The IL-8 mRNA levels were normalized to glyceraldehyde-3-phosphate dehydrogenase (GAPDH) levels. Data were expressed as mean±SEM. *P<0.05 (Mann-Whitney U test), n=4.

Suppression of histone acetylation by blocking of HATs via anacardic acid showed a dose-dependent inhibitory effect on LPS-stimulated IL-8 production ( Figure 6), indicating that histone deacetylation regulates IL-8 expression in LPS-treated epithelial cells. The effects of anacardic acid on IL-8 mRNA expression were analyzed by qRT-PCR. Anacardic acid at 100μM administered 1 h before LPS (10μg/ml) stimulation showed a significant inhibitory effect on LPS-induced IL-8 mRNA levels ( Figure 7). These observations indicated that modulation of histone acetylation by anacardic acid regulated LPS-stimulated IL-8 gene expression in A549 cells.

Raw data for figures 1-7: Anacardic acid, a histone acetyltransferase inhibitor, modulates LPS-induced IL-8 expression in a human alveolar epithelial cell line A549. Figure 1: The dose- and time- dependent IL-8 release from A549 cells in vitro.
Figure 2: Time course analysis of LPS-mediated IL-8 gene expression in A549 cells in vitro.
Figure 3: Time course analysis of LPS-induced histone H4 acetylation at the promoter region of the IL-8 gene in A549 cells in vitro .
Figure 4: Effect of TSA on LPS-stimulated IL-8 release from A549 cells in vitro.
Figure 5: Time course analysis of TSA on LPS-stimulated IL-8 gene activation from A549 cells in vitro.
Figure 6: Effect of anacardic acid on LPS-stimulated IL-8 release from A549 cells in vitro.
Figure 7: Time course analysis of anacardic acid on LPS-stimulated IL-8 gene activation in A549 cells in vitro.

## Discussion

In the present study, we demonstrated that *E. coli*-derived LPS stimulate human alveolar epithelial A549 cells to express and release IL-8, an important chemokine for the local recruitment of neutrophils as an initial defense mechanism ^8, 9^. The specific histone acetylation processes were evaluated by ChIP assay, which clearly showed that LPS induced histone H4 acetylation in A549 cells. Next, we studied the effects of TSA, a HDAC inhibitor on LPS-induced IL-8 expression. TSA showed a significant stimulatory effect on IL-8 production, whereas this agent tended to increase, although not significantly, IL-8 mRNA levels in LPS-stimulated A549 cells. Finally, a HAT inhibitor, anacardic acid, significantly decreased IL-8 mRNA levels as well as protein release in a dose-dependent fashion. These results suggested that LPS-induced IL-8 gene expression is, at least in part, regulated by histone H4 acetylation/deacetylation balance at the IL-8 promoter region.

It has been reported that alveolar epithelial cells respond to bacterial products such as LPS to produce a variety of inflammatory cytokines and mediators including IL-8 ^2^. We ^23, 24^ and others ^9, 25, 26^ have previously shown that airway and alveolar epithelial cells, including A549 cells, are activated by a variety of endogenous agents such as cytokines as well as exogenous stimuli including LPS and fine particles, and express biologically active compounds including cytokines and chemokines.

The transcription of many genes is known to correlate with levels of acetylated nuclear histone proteins ^14– 16^. The expression of many inflammatory genes involves the remodeling of the chromatin structure provided by histone proteins. Histone acetylation causes the unwinding of the chromatin structure, thereby enabling transcription factors to bind to their specific promoter sites on the DNA. Such acetylation processes are reversible and regulated by HATs, which promote acetylation, and HDACs, which promote deacetylation ^18, 19^.

Proinflammatory gene transcription regulation is a multifaceted process that requires integrated sequential molecular events for maximal gene transcription to occur. Activation of specific gene expression needs to be associated with co-operated chromatin remodeling by histone acetylation and binding of transcription factors to their specific binding sites on DNA. In the present experiments, the levels of histone H4 acetylation peaked 1 h after LPS stimulation followed by the maximal levels of IL-8 mRNA at 2 h; such a time course was consistent with the above scenarios.

Our study supports a hypothesis that histone H4 acetylation could play a key role in inflammatory gene transcription such as IL-8 in A549 cells caused by LPS; however, the role of acetylation of the other histones in this model is yet to be established. It has been reported that a variety of histone acetylations are involved in cytokine transcriptional processes. Miyata et al. ^27^ have shown that H3 and H4 are acetylated in murine epithelial cells in response to granulocyte-colony stimulating factor (G-CSF) at the myeloperoxidase gene promoter site, a response dependent on MAPK activation.

Anacardic acid is a bioactive phytochemical found in the nutshell of *Anacardium occidentale* ^28^. The promising effect of anacardic acid on IL-8 gene regulation in the current study has already been reported by Schmeck and associates ^29^ in *Legionella pneumophila*-derived flagellin-induced IL-8 expression. Although the available records are promising, more detailed investigation into the therapeutic properties of anacardic acid, particularly the anti-cancer and anti-inflammatory activities, are needed ^30^.

LPS-mediated IL-8 production is generally expected to act as a defense mechanism which recruits neutrophils in the local environment and facilitates bacterial elimination. However, it is now also known that excessive accumulation of activated neutrophils in the lung is likely to cause excessive inflammatory changes that damage lung tissues ^8^. In this context, appropriate control of local inflammation would be an important strategy for the management of severe pneumonia ^22^. Recently, it has been attracting attention that macrolide antibiotics added to guideline-based therapy has shown a better outcome than respiratory quinolones among intensive care unit (ICU) hospitalized patients with severe pneumonia and/or sepsis ^31^. It has been speculated that certain anti-inflammatory actions of macrolides were involved in such clinically beneficial effects ^32^. Therefore, the suppressive effects of anacardic acid on IL-8 gene expression and production might become a novel strategy for controlling excessive inflammation in order to lessen the risks of acute lung injury and, ultimately, respiratory failure to death.

In conclusion, we have shown that *E. coli*-derived LPS-induced expression and release of IL-8 are associated with increased acetylation of histone H4 expression. The HDAC inhibitor TSA increased IL-8 release. In contrast, a HAT inhibitor, anacardic acid, significantly decreased IL-8 mRNA levels as well as protein release in a dose-dependent fashion. These results suggested that regulation of HDAC and HAT activity by low molecular weight agents might become a novel strategy for the appropriate control of inflammation by modulating inflammatory mediators such as IL-8.
