# Supplementary material for: Anacardic acid, a histone acetyltransferase inhibitor, modulates LPS-induced IL-8 expression in a human alveolar epithelial cell line A549
Source: F1000Res. 2013 Mar 6;2:78. [Version 1] doi: 10.12688/f1000research.2-78.v1 (PMC3931454; doi:10.12688/f1000research.2-78.v1)
Supplement: Peer review history

# Peer review: Referee response for version 1

**Published:** 2013-03-11

**Authors:** Shu Hashimoto

**Affiliations:** 1 The Department of Respiratory Medicine, Nihon University School of Medicine, Tokyo, Japan

This manuscript is well written and conducted. The data and conclusions are clear and worthwhile for the readers.

I have read this submission. I believe that I have an appropriate level of expertise to confirm that it is of an acceptable scientific standard.

# Peer review: Referee response for version 1

**Published:** 2013-03-07

**Authors:** Bruce Rubin

**Affiliations:** 1 Department of Pediatrics, Children’s Hospital of Richmond, Richmond, VA, USA

This is an extremely well conducted study from a top research group. Takizawa and colleagues clearly demonstrate the role of histone acetylation in airway epithelial cell signal transduction after exposure to bacterial endotoxin. These findings are novel and may help in identifying novel targets for modulating chronic airway inflammation.

I have read this submission. I believe that I have an appropriate level of expertise to confirm that it is of an acceptable scientific standard.
